# How Should Debriefing Be Undertaken in Web-Based Studies? Findings From a Randomized Controlled Trial

**DOI:** 10.2196/jmir.2186

**Published:** 2012-11-16

**Authors:** Jim McCambridge, Kypros Kypri, Amanda Wilson

**Affiliations:** ^1^Department of Social & Environmental Health ResearchFaculty of Public Health & PolicyLSHTM, LondonLondonUnited Kingdom; ^2^Centre for Clinical Epidemiology & BiostatisticsSchool of Medicine and Public HealthUniversity of NewcastleCallaghan NSW 2308Australia

**Keywords:** ethics, debriefing, deception, online, web-based, randomised controlled trial, methodology

## Abstract

**Background:**

Internet research may raise older ethical issues in new forms or pose new issues. It has been recommended that debriefing information online be kept very short, with further information including study results made available if requested by participants. There are no empirical studies that compare possible alternative methods of debriefing in online studies.

**Objective:**

To undertake a randomized controlled trial evaluating how to implement the recommended approach by assessing the effects of two different approaches on accessing of additional information.

**Methods:**

All 11,943 participants in the Effects of Study Design and Allocation (ESDA) study, which employed deception, were randomly assigned to one of two methods of debriefing: Group A received the debriefing information in the body of an email with links to protocol and results pages; Group B was presented with these links after clicking on an initial link in the body of the email to view the debriefing information on a website. Outcomes assessed were the proportions clicking on the links to the protocol and results summary and the time spent on these pages by those accessing them.

**Results:**

The group who were presented with no debriefing information in the body of the email and went to a website for this information (Group B) were approximately twice as likely to subsequently access the protocol and the results summary. These differences between the two groups were highly statistically significant. Although these differences are clear, the overall proportions accessing such information were low, and there were no differences in mean time spent reading these pages. Only one quarter of Group B actually accessed debriefing information.

**Conclusions:**

In circumstances where the uptake of fuller information on study design, methods, and findings is deemed important, debriefing information may be better provided via a link and not included in the body of an email. Doing so may, however, reduce the extent of receiving any debriefing information at all. There is a wider need for high quality empirical studies to inform ethical evaluations.

**Trial Registration:**

Australian New Zealand Clinical Trials Registry, ACTRN12610000846022 (http://www.anzctr.org.au/)

## Introduction

Behavior change interventions for public health purposes, as with health care itself, are increasingly being delivered and evaluated using the Internet [1-3]. This development may pose existing ethical questions in new forms, or pose new questions [4]. For example, it may be difficult to know whether research participants actually read study information and give genuinely informed consent. The adequacy of informed consent may also be difficult to assess in non-Internet studies [5], and dedicated investigations typically find that recall of consent is poor and may benefit from intervention [6]. Because of these challenges, ethical guidance for behavioral research on the Internet has been produced [2,7]. Among the recommendations are that debriefing information be kept very short, with further information including study results made available if requested by participants [7]. We are unaware of any empirical studies that compare possible alternative methods of debriefing in online studies. Such data would assist evaluation of ethical issues relating to debriefing.

The Internet is also a useful vehicle for methodological research on participant behavior, partly by virtue of the direct access to large numbers of study participants it affords. Blinding is recommended by the Cochrane Collaboration [8] and others as a means of constraining bias in intervention research. Social psychological research routinely uses deception for similar methodological reasons (for a review see [9]), although the use of deception has not been studied very much in relation to health [10]. Ethical guidance usually requires debriefing following deception [11] though the content of methods of debriefing has been neither widely considered nor studied [12]. Debriefing involves giving study information after the study has ended that would usually be provided prior to participation to permit informed consent. This is usually accompanied with a brief explanation of the rationale for the study design and where there is any potential for harm this can be explored.

This study is a randomized controlled trial evaluating how to implement the recommended approach to debriefing by assessing the effects of two different methods on participants’ accessing of additional information indicative of successful engagement with debriefing.

## Methods

We have previously undertaken a methodological study, the ESDA trial, investigating the possible effects of study design and allocation on participant behavior in the context of a study appearing to investigate alcohol consumption [13]. Almost 12,000 students from four universities in New Zealand participated and were randomized to one of three study conditions, which differed only in what the participants were told was the nature of the study and their role in it. One group believed they were participating in a cohort study, while the other two groups believed they were participants in the intervention and control groups respectively in a randomized controlled trial evaluating an alcohol education intervention, to which all three groups were given access [13]. After the collection of one month outcome data, for the present study we further randomized all participants to two alternative forms of debriefing.

All ESDA participants were randomly allocated to either Group A or Group B. Randomization was computerized and stratified by university, so that there were not imbalances in allocations within any of the 4 participating universities. This and all other study procedures were fully automated and could not be subverted. Allocation was thus fully concealed. Both Groups received an email, sent out on September 22nd, 2011. The initial contents of the emails for both groups are provided in Textbox 1. We allowed 6 weeks for students to respond to the emails, terminating the study on November 11th, 2011. Group A received the debriefing information presented in Textbox 2 in the body of the email, after the text provided in Textbox 1, with links to the protocol (on the journal website) and results pages (see Textbox 3) via [14]. Group B received an email containing no debriefing information, with links to the protocol and results via [15] where the basic debriefing information in Textbox 2 was presented. Group B thus looked at the debriefing information after clicking on a link to the website rather than in the body of the email. All available trial outcome data comprised the proportions clicking on the links to the protocol and the results summary in both groups and the time spent on these pages among those accessing them. We are also able to report on the proportion of Group B accessing the debriefing information on the website and on the time spent reading this page. We tested differences between groups in chi-squared tests for the former and Kruskal-Wallis tests for the latter, for which we report medians. The latter statistical test was chosen in light of the observed gross non-normality with some participants spending very little time with the pages open and others spending more time reading. A non-parametric test was judged preferable to a parametric test to analyze this distribution.

**Initial Email Contents for Both Groups.**

**Group A**

**Subject Line: Tertiary Student Health Project - Information for Participants**

 

In 2010/11 you participated in an online survey about student drinking. Thank you for taking part. As promised, we would like to provide you with some more information about this study which is available below.

This is the last email you will receive about this study. Our database containing participant email addresses will now be deleted.

The iPads were won by students from University of Otago and Victoria University Wellington.

 

**Group B**

**Subject Line: Tertiary Student Health Project - Information for Participants**

 

In 2010/11 you participated in an online survey about student drinking. Thank you for taking part.

As promised, we would like to provide you with some more information about this study which is available here.

<LINK)

This is the last email you will receive about this study. Our database containing participant email addresses will now be deleted.

The iPads were won by students from University of Otago and Victoria University Wellington.

If you experience problems with this link, please copy and paste the link into a new window.

**Basic Debriefing Text in Body of Email for Group a Only, Accessed Via Link for Group B.**

The study randomly assigned people to one of three groups (A, B or C). Group A was told they were completing two surveys. Group B was told they were in a Control Group in a randomised controlled trial evaluating brief alcohol education. Group C was told they were in an Intervention Group in the same trial. In fact, all participants received the same information about alcohol. Apart from what they were told about the nature of the study, there were no differences between the groups. Any differences in reported alcohol consumption were expected to be due to the type of research study people thought they were involved in.

It is unknown whether people change their drinking behaviour, or their reporting of it, according to what type of study they are in. This was worth knowing because it has implications for how research on drinking and other behaviours is conducted and interpreted. We did not find any differences between any of the groups.

As stated in the Information Sheet, no individually identifying information has been collected and your anonymity has been preserved throughout the study.

**Results summary (available to both Group A and Group B).**

In this study we tested two hypotheses:

That knowledge of participation in a randomised controlled trial in comparison to a cohort (ie, before and after surveys) study alone will reduce drinking after 1 month. This was tested by comparing Group A versus Groups B and C together.

That knowledge of allocation to an intervention condition in comparison to a control condition in a randomised controlled trial will reduce drinking after 1 month. This was tested by comparing Group B versus Group C.

Both hypotheses were rejected, as no differences were found between Groups A, B and C. This means the type of study people believed they were in did not influence changes in their drinking behaviour or their reporting of it.

We interpreted the implications of each of the findings for the two hypotheses differently because of the way this study was conducted. In relation to hypothesis 1, it may be worth generating a stronger sense of being in a randomised controlled trial in a future study. We do not believe hypothesis 2 needs further testing.

## Results

**Figure 1:**
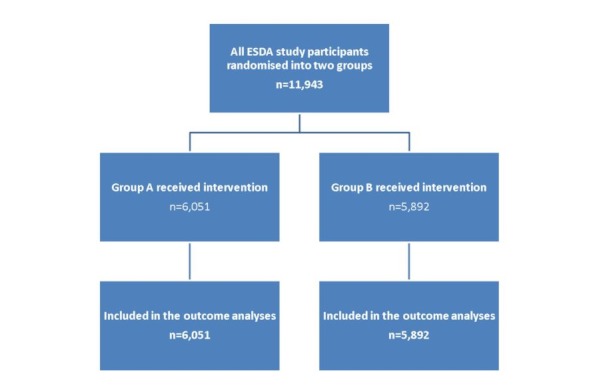
CONSORT flowchart.

The CONSORT flowchart summarizing the study design and numbers included in the analyses is presented in Figure 1.

Group B was approximately twice as likely to have clicked on the protocol and results links, although the proportion doing so was less than 10% in each case (see Table 1). Group B was not likely to spend any more time reading this material. Approximately one quarter of this group visited the debrief page (see Table 1) and thus accessed any debriefing information at all, however, and we had no capacity to measure the extent of any reading of the debriefing information provided in the body of the email to Group A. Approximately one third of those who visited the debriefing page in Group B subsequently clicked on the link for results, and approximately one quarter did so for the protocol (see Table 1).

**Table 1:** All outcome data.

		Group A (N = 6051)	Group B (N = 5892)
**Clicked on Results link**		247 (4.1%)^a^	515 (8.7%)^a^
	Median time in seconds (interquartile range)	25.0 (37.2)^b^	25.3 (35.8)^b^
**Clicked on Protocol link**		202 (3.3%)^a^	362 (6.1%)^a^
	Median time in seconds (interquartile range)	3.5 (5.4)^c^	3.6 (3.9)^c^
**Visited Debrief page**		^d^	1427 (24.2%)
	Median time in seconds (interquartile range)	^d^	36.1 (49.2)

^a^ *P* values for differences between groups in chi-squared tests are all <0.001.

^b^ *P* value for difference between groups from Kruskal-Wallis test = 0.7419.

^c^ *P* value for difference between groups from Kruskal-Wallis test = 0.9450.

^d^ Not applicable to group A.

## Discussion

There are two main sets of findings to consider. First, the between-group differences in the trial demonstrate that providing a link to basic debriefing information (rather than doing so within the body of an email message) approximately doubles the probability that participants will access further debriefing information by clicking on follow-on links. Secondly, the low overall levels of accessing this further information warrant consideration as does the low level of receipt of any debriefing information provided on a website (for Group B) rather than in the body of an email (as for Group A). The robustness of these findings is considered prior to assessment of their ethical and methodological implications.

The messages were all delivered to the email addresses of students held by the universities and via which students participated in the ESDA trial. In the CONSORT flowchart (see Figure 1), we have defined intervention receipt as dispatch of email. We assume that almost all were received and almost all were opened; however, we have no confirmatory data. Some students may have ignored the messages or were no longer using their university email address. We also cannot know for certain how much of the text presented in Textbox 2 was actually read by those who opened the email (Group A) or visited the web page (Group B). Strictly speaking, our time spent on each page measure is of how long the page is open. It is likely that not all of this time was spent actually reading text if other distractions occurred; however, this should have been equally likely in both groups. A clear strength of the data reported here is that they are not reliant on self-report and subject to reporting biases, as they are objectively ascertained, both in relation to whether links were clicked and how long the pages were open. We did not measure email reading time, simply because we could not. One-time-only links to web pages were provided to ensure that participants were not counted twice.

It is possible that many more participants read at least some of the debriefing information in the Group A email and were satisfied with this, or otherwise decided that they did not want further information. To maximize the proportion of all participants having at least some debriefing information (for example, where moral accountability to the research participants is deemed most important [12]), putting the information in the body of the email, as was done with Group A, might be preferable. Alternatively, a key purpose of debriefing is to discover and act upon any harms identified. The reactions of those who have been subjected to any form of deception in research are important to consider in ethical evaluations. For example, according to the British Psychological Society, “If this led to discomfort, anger or objections from the participants then the deception was inappropriate”[11]. This was our primary research interest and why we believe that our outcome measures were well chosen: If research participants have concerns raised by online debriefing information, accessing further information is likely to be the first step in addressing these concerns, if it is made easily available. Where the uptake of more detailed information on study design, methods, and findings is deemed important, as may often be the case in studies involving deception, it appears that basic debriefing information could be better provided via a link and not included in the body of an email.

The low levels of access of the basic debriefing information in Group B remain, however, a matter of substantial concern, and they restrict the confidence that one may draw from the effects favoring Group B. Approximately three-quarters of these participants have received no debriefing information at all, and this appears to be a much bigger problem than we would have expected. If debriefing is worth doing, then it should be done as well as possible, in line with the motivating aims of the present study. This is true even in the absence of harms as they are usually conceived, in order to provide moral accountability for the infringement of the right to informed consent [12]. These considerations direct our attention to the initial content of the original emails, shown in Textbox 1. It may be worth exploring alterations to this brief text, for example making known the absence of informed consent, in ways specifically designed to encourage the uptake of debriefing information.

In this particular study we chose not to elicit feedback as we have done in other studies because participant willingness to articulate concerns may be compromised if the vehicle provided is to communicate with the investigators. Instead, we checked with the Ethics Committee and with the universities involved and confirmed that they had received no complaints from students concerning any aspect of the research. It is therefore not so straightforward to put the two sets of findings together and determine the most appropriate course of action on debriefing. There is merit in further investigating the uptake rates of any debriefing information observed here for Group B, which have the advantage of reliable measurement. If the present findings are confirmed, on balance, we would judge these uptake rates to be unacceptably low and may prefer instead to include the debriefing information in the body of the email, but we have no means of knowing to what extent there is any engagement with information provided there. Time spent reading the protocol was low. It appears it was largely inspected for a few seconds and then the page closed. The results summary was designed to be brief and can be fully read in around the median time spent with the page open. We cannot know, however, how deeply this information was processed or whether the issues involved were more than superficially considered. Longer reading time would be more encouraging in this regard.

Ethical scrutiny of our own practice is made stronger by the kind of data reported here. Study participants were not exposed to risks or harms beyond the infringement of their rights to informed consent, which we acknowledge is a profound harm in itself. Can we make any assumptions about those who did not read the debriefing information? We believe that we cannot, as it would seem unwise to consider either that they may be unconcerned about the infringement of their right to informed consent as research participants, or alternatively that they would be greatly concerned. There are few previous studies providing helpful data in this regard, though Fisher and Fyrberg [9] identified approximately 70% of university student participants as having a basically utilitarian attitude to infringements of such rights in research and approximately 30% who may be offended, among whom a much smaller proportion were deeply offended. These harms need to be balanced against the research and hence the social value of the data obtained.

In our ethical deliberations on this type of research, we were concerned that debriefing itself may constitute a source of harm by revealing an infringement of rights not previously known. The data in the present study are helpful to ongoing consideration of whether debriefing should be undertaken, as well as how. We found no evidence in this particular study that debriefing itself may be harmful and therefore no reason to discontinue it, although consideration of the cumulative impact of such debriefing, in populations such as students who are regularly invited to participate in research, as well as the use of deception in research more broadly, is warranted. For example, it is entirely possible that those who have been debriefed may be more cautious about participating in future research studies, which would diminish the value of the data obtained in those studies. This type of harm is both important and challenging to evaluate. Empirical data are of course no substitute for ethical reflection; however, they can also undoubtedly enrich it. The paucity of empirical data in relation to ethical decision-making has previously been commented upon [16]. We very much agree there is a wider need for high quality empirical studies, using experimental data in particular, to inform ethical evaluations of future methodological developments, online and elsewhere.
